# Novel Levamisole Derivative Induces Extrinsic Pathway of Apoptosis in Cancer Cells and Inhibits Tumor Progression in Mice

**DOI:** 10.1371/journal.pone.0043632

**Published:** 2012-09-10

**Authors:** Mahesh Hegde, Subhas S. Karki, Elizabeth Thomas, Sujeet Kumar, Kuppusamy Panjamurthy, Somasagara R. Ranganatha, Kanchugarakoppal S. Rangappa, Bibha Choudhary, Sathees C. Raghavan

**Affiliations:** 1 Department of Biochemistry, Indian Institute of Science, Bangalore, Karnataka, India; 2 Department of Pharmaceutical Chemistry, KLE University's College of Pharmacy, Bangalore, Karnataka, India; 3 Department of Studies in Chemistry, University of Mysore, Mysore, Karnataka, India; 4 Institute of Bioinformatics and Applied Biotechnology (IBAB), Bangalore, Karnataka, India; Instituto Nacional de Cardiologia, Mexico

## Abstract

**Background:**

Levamisole, an imidazo(2,1-b)thiazole derivative, has been reported to be a potential antitumor agent. In the present study, we have investigated the mechanism of action of one of the recently identified analogues, **4a** (2-benzyl-6-(4′-fluorophenyl)-5-thiocyanato-imidazo[2,1-b][1], [3], [4]thiadiazole).

**Materials and Methods:**

ROS production and expression of various apoptotic proteins were measured following **4a** treatment in leukemia cell lines. Tumor animal models were used to evaluate the effect of **4a** in comparison with Levamisole on progression of breast adenocarcinoma and survival. Immunohistochemistry and western blotting studies were performed to understand the mechanism of **4a** action both *ex vivo* and *in vivo*.

**Results:**

We have determined the IC_50_ value of **4a** in many leukemic and breast cancer cell lines and found CEM cells most sensitive (IC_50_ 5 µM). Results showed that **4a** treatment leads to the accumulation of ROS. Western blot analysis showed upregulation of pro-apoptotic proteins t-BID and BAX, upon treatment with **4a**. Besides, dose-dependent activation of p53 along with FAS, FAS-L, and cleavage of CASPASE-8 suggest that it induces death receptor mediated apoptotic pathway in CEM cells. More importantly, we observed a reduction in tumor growth and significant increase in survival upon oral administration of **4a** (20 mg/kg, six doses) in mice. In comparison, **4a** was found to be more potent than its parental analogue Levamisole based on both *ex vivo* and *in vivo* studies. Further, immunohistochemistry and western blotting studies indicate that **4a** treatment led to abrogation of tumor cell proliferation and activation of apoptosis by the extrinsic pathway even in animal models.

**Conclusion:**

Thus, our results suggest that **4a** could be used as a potent chemotherapeutic agent.

## Introduction

Cancer is a difficult disease to treat, and only very few effective drugs are available. The development of novel, efficient, selective and less toxic cancer therapeutic molecules has been a challenging goal. Understanding the molecular mechanism involved in cancers will lead to the discovery of novel anticancer agents. Changes in expression levels of RNA and proteins due to different mutations have been studied in many cancers, including leukemia and lymphoma [1]–[4]. Recently, there have been extensive efforts to characterize the mechanism of chromosomal translocations and deletions resulting in leukemia and lymphoma [5], [6]. Many gene fusions have also been identified in prostate cancers and breast cancers [7]. The most discussed proteins responsible for leukemia and lymphoma in the recent past are the recombination activating genes (RAGs, the enzyme responsible for antibody diversity) [5], [6] and activation induced deaminase (AID, the enzyme responsible for somatic hypermutation and class switch recombination) [5], [8]. However, the enzymes responsible for the development of gene fusions are yet to be identified.

The past two decades have seen a dramatic change in cancer treatment paradigms. For example, Imatinib (Gleevac), a drug developed specifically against the activated tyrosine kinase in chronic myelogenous leukemia, is one of such major advances [9]. In addition, many other compounds have also been identified and clinically tested. Although, the success of clinical trials in identifying new agents and treatment modalities has been significant, the current treatments have many limitations. This includes side effects induced by the drugs and acquired drug resistance [10]. Thus, the need for the development of effective anti-cancer therapeutic agents with well-defined pharmacokinetic properties is of great importance.

Currently, there are different ways by which a drug is tested for its effectiveness as an anticancer agent. In this regard, various apoptotic pathways have been studied extensively for many compounds to understand their mode of cytotoxicity [11]. Cell cycle check points induced by small molecules have also been investigated [12], [13].

Levamisole is an immunomodulator in different cancer cells including colorectal, breast cancer, melanoma, and leukemia [14]. Previously, it has been shown that it affects cell proliferation in different cancers [15] and modulates the phosphorylation relevant for both cell cycle progression and apoptosis. Studies have also shown that it can be used for anti- helminthic infestations and various autoimmune diseases [16], [17]. Besides, it has been shown that levamisole has anticancer activity in combination with fluorouracil (5-FU) as adjuvant therapy for tumor-node-metastasis (TNM) stage III (Dukes' C) colon carcinoma [18].

The imidazo(2,1-b)thiazole derivatives of Levamisole have been reported as potential antitumor agents [19]. Later, antitumor activity of 5-formyl-6-arylimidazo-[2,1-b]-1,3,4-thiadiazole sulfonamides were also reported [20]. Based on these promising results, we synthesized a series of analogues containing fluorine at position 4 of 6-phenyl in imidazo-[2,1-b]-1,3,4-thiadiazole and identified **4a** as the lead compound [21]. However, the mechanism by which it induced cytotoxicity was not known. Besides, it was never tested on animal models for its effect on tumor progression. In the present study, we report that **4a** exerts its effect on tumor cells by activating the extrinsic pathway of apoptosis. We also found that **4a** inhibits the progression of tumor in mice effectively and increases the lifespan significantly.

## Materials and Methods

### Chemicals and reagents

All the chemicals used in the present study were of analytical grade and purchased from Sigma–Aldrich, USA. Antibodies were obtained from Santa Cruz Biotechnology, USA.

### Synthesis of 4a

Synthesis and characterization of 2-benzyl-6-(4′-fluorophenyl)-5-thiocyanato-imidazo[2,1-b][1], [3], [4]thiadiazole, **4a** has been described earlier [21]. Levamisole (Tetramisole hydrochloride, Cat. No. L9756) was purchased from Sigma-Aldrich, USA.

### Cell culture

Human cell lines, CEM (T-cell leukemia), K562 (Chronic myelogenous leukemia) REH (B-cell leukemia) and Nalm6 (B-cell leukemia), were cultured in RPMI1640 (Sera Lab, UK) containing 10% FBS (Gibco BRL, USA), 100 U of Penicillin G/ml and 100 µg of streptomycin/ml (Sigma–Aldrich, USA) at 37°C in a humidified atmosphere containing 5% CO_2_. EAC (breast cancer) cell line was purchased from National Center for Cell science, Pune and grown in DMEM containing 10% FBS as described above.

### Trypan blue dye exclusion assay

The effect of **4a** on viability of leukemic (CEM, K562, REH, Nalm6) and breast cancer (EAC) cells were determined by Trypan blue dye exclusion assay [22]. Cells were cultured (0.75×10^5^ cells/ml) for 24 h and compound was added in the range of 1–100 µM to determine the IC_50_ value. DMSO treated cells were used as vehicle control. Cells were collected at intervals of 24 h for five days and number of viable cells was determined following trypan blue staining. For Levamisole, water was used as vehicle control. In case of EAC, an adherent cell line, viability was measured at 48 and 72 h after treatment of **4a**. Each experiment was repeated a minimum of two times and error bars were calculated and plotted.

### MTT assay

The MTT assay was performed as described earlier [23]. CEM, K562, REH or Nalm6 cells (0.75×10^5^ cells/ml) were treated with **4a** (for CEM and REH cells 1, 5, 10 and 20 µM; for K562 1, 5, 10, 20, 40 and 100 µM; for Nalm6, 1,5,10, 20, 40 µM), incubated for 48 and 72 h and subjected to MTT assay. Cells treated with DMSO or water was used as vehicle controls for **4a**, respectively. Experiment was repeated a minimum of two independent times, each with duplicate reactions and the error bars are indicated.

### LDH release assay

LDH release into media following **4a** treatment (1, 5, 10 and 20 µM) on CEM cells after 48 and 72 h of treatment was measured using standard protocol [24]. The percentage of LDH release was calculated as: LDH release in media/(LDH release in media+intracellular LDH release)×100%.

### Detection of intracellular ROS production by flow cytometry

The level of total intracellular ROS production was measured by using cell permeable fluorescent probe 2,7-dichlorodihydro fluorescein diacetate (H_2_DCFDA) in CEM and REH cells [25]. CEM cells were treated with 5 and 10 µM of **4a** and REH cells with 10 µM for 5, 10, 15, 30 and 60 min, harvested, washed and the fluorescence intensity was analyzed by flow cytometry. Cells treated with H_2_O_2_ were used as positive control for compensation of experimental samples.

### Western blot analysis

Cell lysate was prepared following treatment with **4a** on CEM (0, 0.5, 1, and 5 µM for 48 h). Western blotting was performed as described previously [23]. Briefly, ∼40 µg of protein sample was electrophoresed on 8–12% SDS-PAGE, transferred to PVDF membrane (Millipore, USA) and probed with respective primary and biotinylated secondary antibodies. The primary antibodies used were BCL2, BCL-xL, BAX, t-BID, p53, p-p53 [Ser 392], PUMA, AKT, pAKT [Ser 473], FAS, FAS-L, FADD, SMAC/DIABLO, CASPASE-3, CASPASE-8 and CYTOCHROME C. The blots were developed using chemiluminescent reagents (Immobilon™ western, Millipore, India) and scanned by gel documentation system (LAS 3000, Fuji, Japan). Blots were stripped subsequently as per standard protocol and re-probed with anti-TUBULIN antibody [23].

### Separation of mitochondrial and cytosolic fractions from 4a treated CEM cells

CEM cells were treated with 5 µM **4a** for 48 h, harvested and used for isolation of mitochondrial and cytosolic fractions using mitochondrial extraction kit (IMGENEX, USA, Cat. No. 10082k) as per the manufacturer's instructions. DMSO treated cells were used as control. The resulting fractions were used for western blot analysis against anti-CYTOCHROME C. Actin was used as loading control.

### In vivo experiments

#### Ethics Statement

Mice were maintained as per the principles and guidelines of the ethical committee for animal care of Indian Institute of Science in accordance with Indian National Law on animal care and use. The experimental design of the present study was approved by Institutional Animal Ethics Committee (Ref. CAF/Ethics/125/2007/560), Indian Institute of Science, Bangalore, India.

### Animals

Swiss albino mice, 6–8 weeks old, weighing 18–22 g were purchased from central animal facility, Indian Institute of Science (IISc), Bangalore, India and maintained in the animal house, Department of Biochemistry, IISc. The animals were housed in polypropylene cages and provided standard pellet diet (Agro Corporation Pvt. Ltd., India) and water ad libitum. The standard pellet diet composed of 21% protein, 5% lipids, 4% crude fiber, 8% ash, 1% calcium, 0.6% phosphorus, 3.4% glucose, 2% vitamin, and 55% nitrogen-free extract (carbohydrates). The mice were maintained under controlled conditions of temperature and humidity with a 12 h light/dark cycle.

#### Preparation of Ehrlich ascites carcinoma (EAC) cells

EAC cells were collected from the peritoneal cavity of tumor-bearing donor mice of 20–22 g body weight and suspended in sterile phosphate buffered saline (PBS). A fixed number of viable cells (1×10^6^ cells/22 g b. wt) were implanted into the peritoneal cavity of each recipient mouse and allowed to multiply. The tumor cells were withdrawn, diluted in saline, counted and re-injected (1×10^6^ cells/animal) to right thigh tissue of experimental animals for developing solid tumor.

### Evaluation of antitumor activity of 4a in mouse

To study and compare the antitumor activity of **4a**, 32 Swiss albino mice were used in the present study, (two batches of 16 animals each). Out of 16 mice, four were used as untreated (normal) control. Rest of the mice were injected with EAC to induce solid tumor, and divided into three groups, each containing four animals for tumor control (group two), Levamisole treated (group three) and **4a** treated (group four). Group two received water as vehicle control, group three received oral administration of Levamisole (20 mg/kg, b. wt) and group four received oral administration of **4a** (6 doses of 20 mg/kg) on every alternative day using gastric gavages starting from 12^th^ day of injection of tumor cells.

The diameters of developing tumor were measured in the case of group two, three and four animals by using vernier calipers once in five days. Tumor volume was calculated using the formula V = 0.5ab^2^, where ‘a’ and ‘b’ indicate the major and minor diameter, respectively [26]. At the end of 25^th^ and 45^th^ day of experimental period, one animal from each group was sacrificed by cervical dislocation and tissues from normal (group one), tumor (group two), Levamisole treated (group three) and **4a** treated (group four) animals were collected and stored.

To check the longevity induced by **4a** in tumor mice, 24 animals were studied, two batches containing 12 each. Out of 12, six served as tumor control and others were treated with **4a** as explained earlier. The percentage of increase in lifespan was calculated and compared with that of control animals. The death pattern for controls and **4a** treated animals was recorded and % increase in lifespan was calculated using the formula [(T−C)/C]×100, where ‘T’ indicates the number of days the **4a** treated animals survived and ‘C’ indicates the number of days tumor animals survived [26]–[28].

### Evaluation of toxicity of 4a in normal mice

Swiss Albino mice were treated with **4a** and Levamisole (6 doses, on every alternate day) and side effects were evaluated at two different time points (20^th^ and 50^th^ day). Out of 36 mice, 18 each were used at 20^th^ and 50^th^ day. In both cases, 6 animals served as control, while 6 were treated with Levamisole (20 mg/kg) or **4a** (20 mg/kg). Body weight of each animal was monitored throughout the experiment and average weight calculated at 20^th^ and 50^th^ day for control, **4a** and Levamisole administered mice and were plotted with error bars. In order to evaluate the effect of **4a** and Levamisole on physiological functions, blood was collected on 20^th^ and 50^th^ day as described earlier [29]. Serum was separated and liver and kidney function tests were performed for each animal, to determine levels of alkaline phosphatase (ALP), creatinine, urea and blood count was carried out using plasma as described earlier [29]. Values are presented as mean±SEM.

### Western blot analysis for 4a treated solid and liquid tumor cells

Solid tumor was developed as described previously [29]. Following 6 doses of **4a**, tumor was collected and extract was prepared using RIPA buffer method [23]. The liquid tumor was developed by injecting EAC cells (2×10^6^ cells) from donor mice to peritoneal cavity of the experimental animals. Following EAC injection (5^th^ day), animals were treated with **4a** (20 mg/kg; 4 doses every alternative days) and EAC cells were isolated from the peritoneal cavity. The macrophage lineage cells were separated from non-adherent EAC cells by gentle aspiration, and washed with 1× PBS. Cell viability was checked using trypan blue dye exclusion assay and 92% cells were found to be alive in both experimental and controls groups. EAC cells were lysed in RIPA buffer, extract was prepared as described earlier [30] and used for western blot analysis.

### Histological evaluation

Tumor and liver tissues of normal and experimental mice were collected and processed as per standard protocols. Briefly, the tissues were embedded in paraffin wax, sectioned at 5–10 µm in a rotary microtome (Leica Biosystems, Germany) and stained with haematoxylin and eosin [31], [32]. Brain tissues were collected, processed and stained with Luxol Fast Blue to study demyelination. Each section was evaluated by light microscopy and images were captured (Zeiss, Germany).

### Immunohistochemical (IHC) analysis

Antibody staining was conducted on formalin fixed, paraffin embedded tissues, which were sectioned at a thickness of 5 µm. Slides were de-paraffinized using xylene, rehydrated and treated with 3% H_2_O_2_ in PBS. Antigen retrieval was done using 0.01% sodium-citrate buffer followed by blocking in PBST containing 0.1% BSA and 10% FBS. Primary antibody incubation (Ki67, BID or 53BP1) was carried out overnight at 4°C. Slides were washed and incubated with biotinylated secondary antibody (1 h). Slides were then washed, incubated in streptavidin-HRP (1∶1000). Slides were again washed (PBS containing 0.1% Tween 20) and colour was developed using DAB+H_2_O_2_, counterstained with haematoxylin and mounted in DPX (Sigma-Aldrich, USA). Images were captured using light microscope (Zeiss, Germany). Change in intensity of antibody staining following **4a** treatment was determined by using ImageJ software [33].

### Statistical analysis

Values are expressed as mean ± SEM for control and experimental samples and statistical analysis was performed using One-way ANOVA followed by Dunnett test and each value was compared with the control and significance is mentioned. For this analysis, GraphPad software prism 5.1 was used. The values were considered as statistically significant, if the p-value was equal to or less than 0.05.

## Results

### 4a induces cytotoxicity in cancer cells

**Figure 1:**
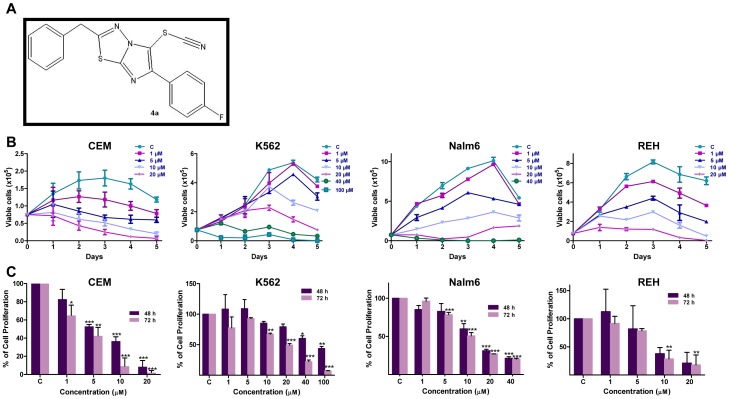
Dose-dependent cytotoxic effect of 4a on leukemic cell lines. **A.** The structure of **4a.** **B.** **4a** induced cytotoxicity as determined by trypan blue assay. CEM, K562, Nalm6 and REH cells were cultured (0.75×10^5^ cells/ml) and cytotoxicity was measured after addition of increasing concentration of **4a** as indicated. Cells were counted at intervals of 24 h until cells attained stationary phase and were plotted. DMSO treated cells were used as vehicle control. Standard error was calculated based on minimum of two independent experiments. **C.** Determination of cell proliferation using MTT assay following addition of **4a** to CEM, K562, Nalm6, and REH cells (48 and 72 h). Results shown are from a minimum of two independent experiments, each was done in duplicates and results are expressed as % of cell proliferation. In all panels “C” stands for DMSO treated vehicle control.

**Figure 2:**
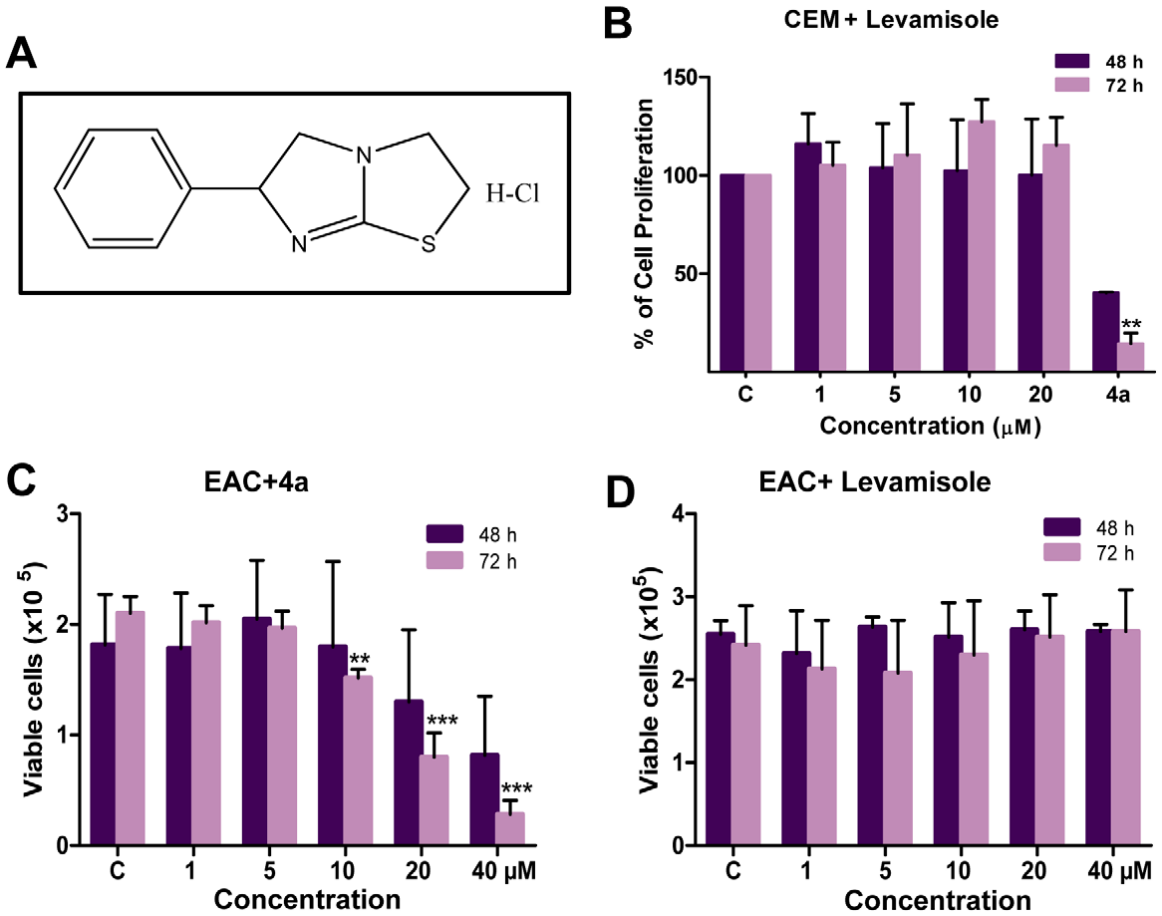
Comparison of cytotoxicity of 4a and Levamisole in CEM and EAC cells. **A.** The structure of Levamisole, the parental compound of **4a**. **B.** Determination of cell proliferation using MTT assay on CEM cells treated with Levamisole or **4a**. In case of Levamisole, concentrations used were 1, 5, 10 and 20 µM, while it was 10 µM for **4a**. Standard error was calculated based on two independent experiments. **C, D.** Cytotoxicity of **4a** and Levamisole on EAC cells as measured by trypan blue assay. EAC cells were cultured (0.75×10^5^ cells/ml) and treated with 1, 5, 10, 20 and 40 µM of **4a** or Levamisole. Viability of the cells were determined by trypan blue assay at 48 and 72 h. Standard error was calculated based on three independent experiments.

Previously, while screening a series of Levamisole derivatives, we identified **4a** as the lead compound (Fig. 1A) [21]. In the present study, we have used a variety of leukemic cell lines (CEM, K562, REH and Nalm6) and a mice breast cancer cell line, Ehrlich ascites carcinoma (EAC) to evaluate its potential to induce cytotoxicity. Firstly, IC_50_ of **4a** on CEM, K562, Nalm6 and REH cells was determined using trypan blue and MTT assays (Fig. 1). Cells treated with DMSO were used as vehicle control. Results showed that **4a** treatment significantly affected cell viability at lower concentrations in CEM, Nalm6 and REH (Fig. 1B, C). Interestingly, K562 cells showed least sensitivity towards **4a** treatment (Fig. 1B, C). Based on both trypan blue and MTT assays, the IC_50_ value was estimated to be approximately 5, 70, 10 and 8 µM in CEM, K562, Nalm6 and REH cells, respectively after 48 h of **4a** treatment. Interestingly, in comparison with **4a**, Levamisole treatment on CEM cells showed less sensitivity (Fig. 2A, B). **4a** exhibited significant cytotoxicity in EAC cells (IC_50_, 33 µM), while cells were insensitive to Levamisole, at the range of concentrations tested (Fig. 2C, D). Further, LDH assay was performed to assay cell damage induced by **4a** on CEM cells. Cells were treated with **4a** for 48 and 72 h, respectively, harvested and subjected to LDH measurement. Results showed a dose-dependent increase in the release of LDH (Fig. S1).

### 4a induces intracellular reactive oxygen species (ROS)

**Figure 3:**
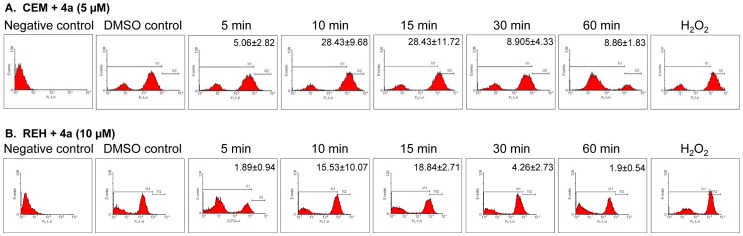
Determination of intracellular ROS production in CEM and REH cells following treatment with 4a. **A, B.** CEM (A) and REH (B) cells treated with **4a** (5 µM and 10 µM, respectively) for different time points were used for testing the formation of intracellular ROS by flow cytometry analysis. The concentration selected for the study was based on their respective IC_50_ values. H_2_O_2_ treated cells were used as positive control while cells alone were used as negative control. DMSO treated cells were used as vehicle control. Cell population showing ROS was shown along with standard error mean (n = 2).

Overproduction of ROS following addition of a compound is an indicator of cellular response leading to DNA damage and apoptosis. We found that **4a** treatment induced ROS production in case of CEM (5 and 10 µM) as well as REH (10 µM) cells at 10 and 15 min (Fig. 3 A,B, Fig. S2). Further, the increase in incubation time did not enhance the ROS level. Cells treated with H_2_O_2_ were used as a positive control, while DMSO treated cells served as vehicle control (Fig. 3). Thus, our results suggest that ROS production is an intermediate step involved in **4a** induced cytotoxicity.

### 4a modulates expression of apoptotic proteins

**Figure 4:**
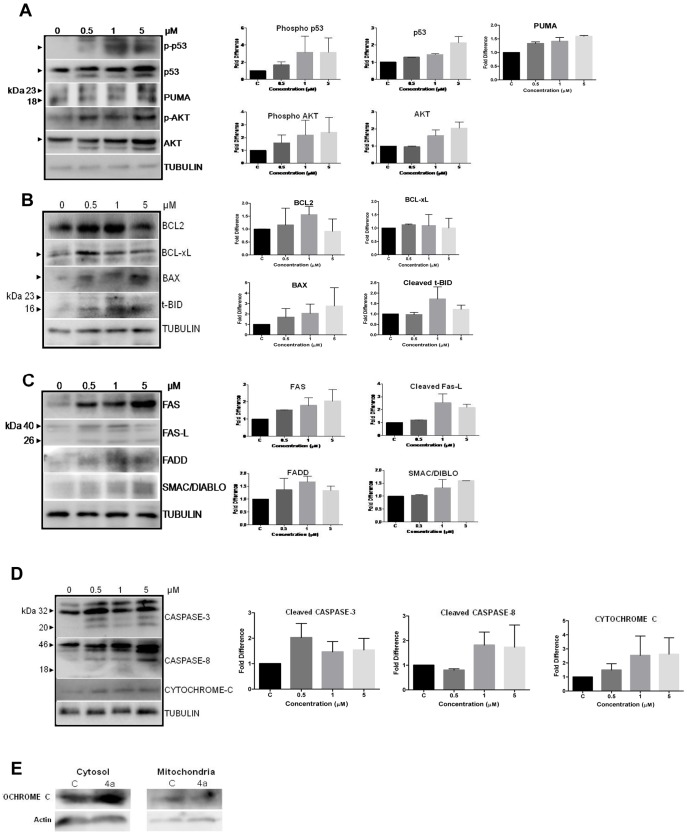
Expression of apoptotic proteins in CEM cells after 4a treatment. CEM cell lysate was prepared following treatment with **4a** (0, 0.5, 1 and 5 µM for 48 h). DMSO treated cells were used as control (0 µM). Western blotting studies were performed using specific primary and secondary antibodies for expression of (**A**) Phospho p53, p53, PUMA, phospho AKT, AKT (**B**) BCL2, BCL-xL, BAX and t-BID; (**C**) FAS, FAS-L, FADD, and SMAC/DIABLO (**D**) CASPASE-3, CASPASE-8 and CYTOCHROME C. α-TUBULIN was used as loading control. The quantification of the bands in each blot shown in left panel is shown as bar diagram with standard error based on two independent experiments following normalization with respective TUBULIN **E.** Release of CYTOCHROME C from mitochondria after treatment with **4a**. Mitochondrial as well as cytosolic fractions were separated from CEM cells after 48 h of treatment with **4a** (5 µM), DMSO treated cells were used as control (C), western blotting was performed using anti-CYTOCHROME C. Actin was used as loading control.

In order to study the mechanism by which **4a** induces cell death, we studied the expression levels of different apoptotic proteins following **4a** treatment. CEM cells were chosen for the study as it showed the maximum sensitivity to **4a**. CEM cells were treated with increasing concentrations of **4a** (0.5, 1 and 5 µM, for 48 h), cell lysate was prepared and used for western blot studies. Results showed that **4a** treatment led to a remarkable increase in the levels of p53 as well as phospho-p53 (Fig. 4A). Since p53 is a known activator of apoptosis, we tested the expression of various BCL2 family proteins which have pro/antiapoptotic functions (Fig. 4A, B). Consistent with our above results, we observed an increase in the expression of proapoptotic proteins PUMA, BAX, and cleavage of BID (Fig. 4A, B). Interestingly, we also observed the upregulation of antiapoptotic proteins, BCL2 and BCL-xL, particularly at 0.5 and 1 µM concentrations (Fig. 4B). Further, **4a** treatment led to the increase in expression of death-receptor signaling proteins, FAS, FAS-L and FADD, indicating that cytotoxicity induced by **4a** could be mediated through the death receptor mediated apoptosis (Fig. 4C).

PI3K/AKT pathway is known to be activated in a majority of T-ALL. It is also known that it plays a critical role in controlling survival and apoptosis. Increase in p-AKT shifts the cells towards survival by interfering with p53 mediated pathway of apoptosis. Hence, we were interested in checking the levels of AKT after compound treatment. Results showed upregulation of AKT following addition of **4a** (Fig. 4A). Inspite of increase in the levels of p-AKT, the drug induced cell death, which suggests that the ratio of proapoptotic and antiapoptotic signals in the cell was disrupted.

SMAC/DIABLO is a mammalian mitochondrial protein that functions as a regulatory component during apoptosis. We tested its expression upon **4a** treatment and results showed an upregulation of the protein expression (Fig. 4C). A dose-dependent increase in the level of CYTOCHROME C was also observed (Fig. 4D) [34]. We also checked for the release of CYTOCHROME C to cytoplasm and results showed a distinct increase in the level of the cytosolic CYTOCHROME C upon treatment with **4a** (Fig. 4E).

CASPASE-8 is another protein activated during the extrinsic pathway of apoptosis. Results showed cleavage of CASPASE-8 upon **4a** treatment (Fig. 4D). This further confirms the activation of the death-receptor mediated apoptosis. Activated CASPASE-8 also cleaves PROCASPASE-3 and consistent with this we find activation of PROCASPASE-3 compared to the controls, upon **4a** treatment, in a dose-dependent manner (Fig. 4D).

### 4a treatment inhibits tumor progression in mice

**Figure 5:**
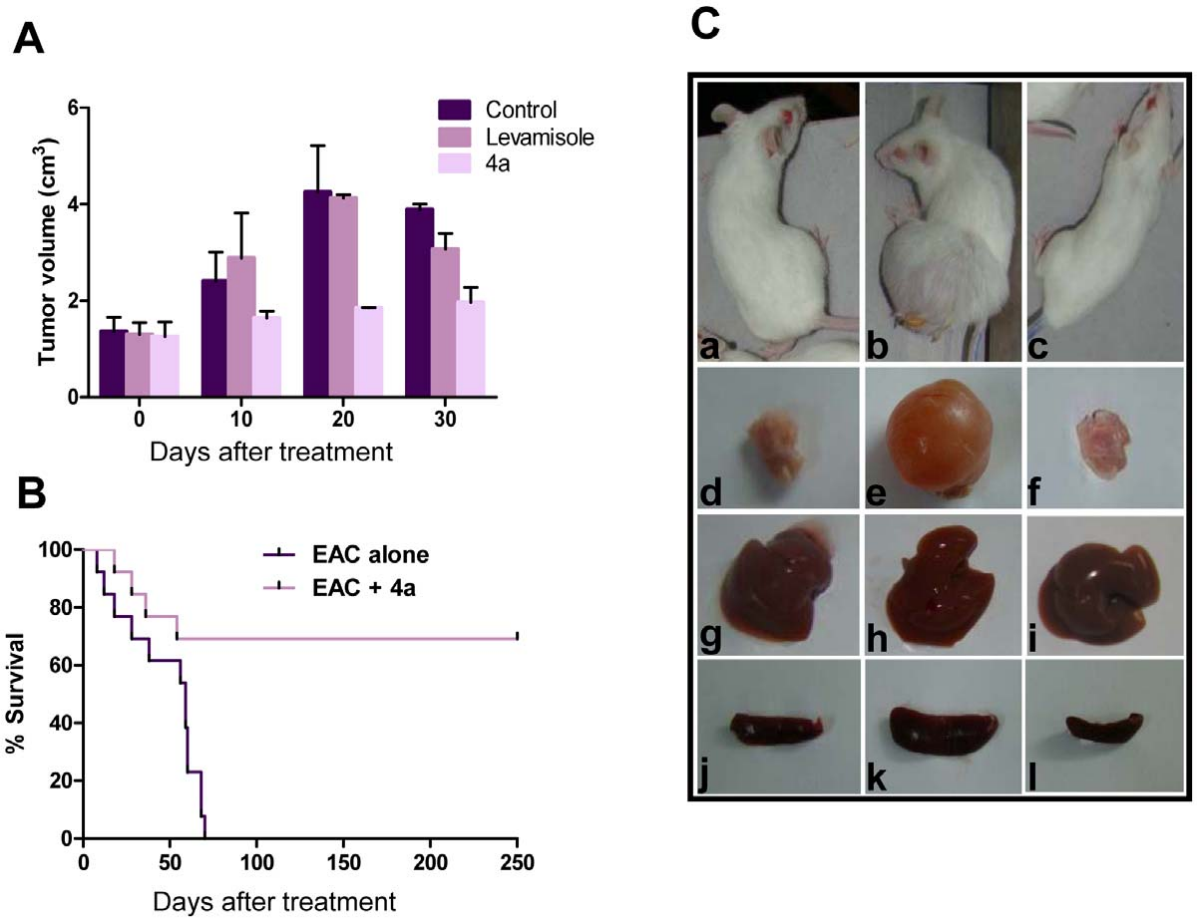
Comparison of effect of 4a and Levamisole on progression of solid tumor in mice. Solid tumor was induced in Swiss albino mice by injecting EAC cells. Six doses of **4a** and Levamisole (20 mg/kg) each administered to tumor bearing mice on every alternate day from 12^th^ day of EAC cell injection. **A.** Effect of **4a** and Levamisole on tumor progression at different time points. Data shown is based on two independent batches of experiments containing four animals each. Error bars indicate SD from independent experiments. **B.** Kaplan–Meier survival curves of mice treated with **4a**. Out of 24 tumor induced Swiss Albino animals, 12 were treated with **4a** (20 mg/kg) and survival graph was plotted, Log-rank statistical test showed P<0.005 (**). In control case, median survival time was found to be 59 days and in case of 4a treated it is undefined (value showed up to 250 days). **C.** Gross appearance of **4a** treated and untreated tumor mice and their selected organs at 25^th^ day of treatment. **a.** mouse with no tumor, **b.** mouse bearing tumor, **c.** tumor bearing mouse after treatment with **4a**, **d**. thigh tissue of normal mouse, **e.** tumor, **f.** thigh tissue of a treated mouse, **g.** liver from normal mouse, **h**. liver of a tumor mouse, **i.** liver from a **4a** treated mouse, **j**. spleen of a normal mouse, **k.** spleen of a mouse with tumor, **l.** spleen of a treated mouse.

EAC derived from breast adenocarcinoma is an aggressive and rapidly growing carcinoma commonly used for the evaluation of the effect of novel small molecules on tumor progression. Based on pilot studies, 20 mg/kg body weight of **4a** was used for treatment in animals bearing tumors (data not shown). After 12^th^ day of EAC injection (small size tumor was visible), the animals were treated with six doses every alternate day. We found that treatment with **4a** on animals bearing tumor resulted in significant reduction of tumor size compared to that of untreated as well as Levamisole treated tumor animals (20 mg/kg) (Fig. 5A). We found that 80% of the mice survived upon treatment with **4a**, whereas 50% of the untreated tumor mice were dead between 30 to 40 days of tumor development (Fig. 5A, B and data not shown). The gross appearance of thigh tissue containing tumor, liver and spleen of negative control, untreated tumor control and **4a** treated mice showed a proportional morphological difference (Fig. 5C and data not shown).

More importantly, we found that upon treatment with **4a**, animals with tumor showed a significant difference in the survival rate compared to the untreated tumor control (Fig. 5B). While control animals survived for only a maximum of 70 days after tumor development, majority of mice treated with **4a** survived for more than 250 days indicating a ∼4-fold increase in life span (Fig. 5B). Therefore, our results demonstrate that **4a** treatment significantly reduced the tumor load and increased the lifespan of the animals.

Histological evaluation was also performed at two different time points (25^th^ and 45^th^ day) of treatment. Sections from tumor tissue of a 25 day treated mouse showed many haematoxylin stained nuclei with little cytoplasmic staining indicating active cell proliferation, while in the case of controls, no other cells other than the nuclei of skeletal muscles were stained (Fig. S3A). **4a** treated tumor tissues showed a significant reduction in proliferating cells (Fig. S3A). Tissue sections from thigh after 45^th^ day of treatment showed negligible number of proliferating cells and were more comparable with that of normal tissues, while proliferating cells were abundant in mice bearing tumor, where no treatment was given (Fig. S3A). To analyze whether **4a** treatment had any adverse effect on other tissues, sections of liver were analyzed by haematoxylin and eosin staining (Fig. S3C,D). Our results showed hypertrophy of hepatocytes in both tumor bearing and **4a** treated mice. However, it was restored back to normal only in cases where the tumour regressed after treatment with compound **4a**, unlike the untreated mice where irregular hepatocytes were still seen (Fig. S3C, D). Thus, our results show that **4a** could be used as a potent anticarcinogenic agent.

### The effect of 4a on normal mice

**Figure 6:**
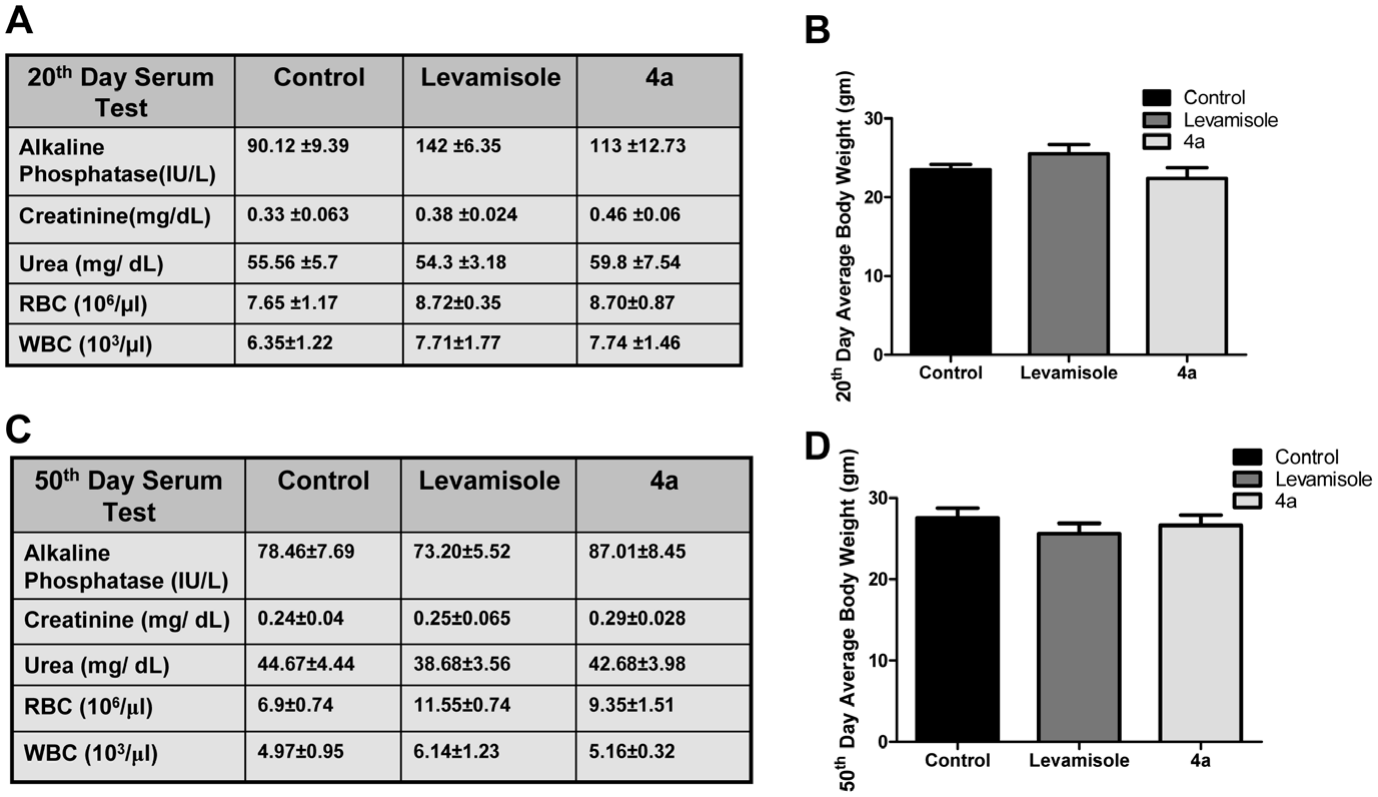
Evaluation of side effects of Levamisole and 4a in Swiss Albino mice. **4a** or Levamisole were orally administered (20 mg/kg, six doses in interval of two weeks) to experimental animals and body weight was monitored on 20^th^ or 50^th^ day, blood was collected and serum was checked for alkaline phosphatase (ALP), creatinine; urea, and plasma was used for counting RBCs and WBCs to analyze the side effects. **A, C.** Evaluation of kidney and liver function following 20 and 50 days, respectively, of **4a** treatment. **B, D.** Assessment of body weight changes in mice following 20 and 50 days after **4a** and Levamisole treatment. Value of serum tests and blood counts are given with mean±SEM (n = 6), average body weight of each group was plotted with standard error.

It was important to study the side effects of **4a**, as its parental analogue Levamisole showed variety of side effects in animals as well as human beings [35], [36]. To assess the side effects of **4a** and Levamisole, it was orally administered to normal mice as described in methods. Results showed significant increase in alkaline phosphatase (ALP) level in case of Levamisole treated mice (∼50% increase compared to control) after 20 days of treatment. Unlike, Levamisole, **4a** showed only ∼20% increase in ALP levels (Fig. 6A). The liver sections also showed a similar effect (Fig. S4A). Besides, kidney function tests for creatinine, urea also showed comparable levels as in controls upon **4a** treatment. WBC, RBC counts and body weight were also found to be normal compared to control in **4a** and Levamisole treated cases (Fig. 6A, B). Brain tissues were subjected to Luxol Fast Blue staining to check the status of myelination. Results suggested that both the molecules were nontoxic to the brain at the used concentration and doses. Interestingly, 50^th^ day post treatment showed normal ALP level in serum in both the cases suggesting that local toxicity in liver showed by both the molecules were transient and could be recovered with time (Fig. 6C, D).

### Treatment with 4a leads to reduction in proliferating cells while expression of apoptotic proteins increases in tumor tissues

**Figure 7:**
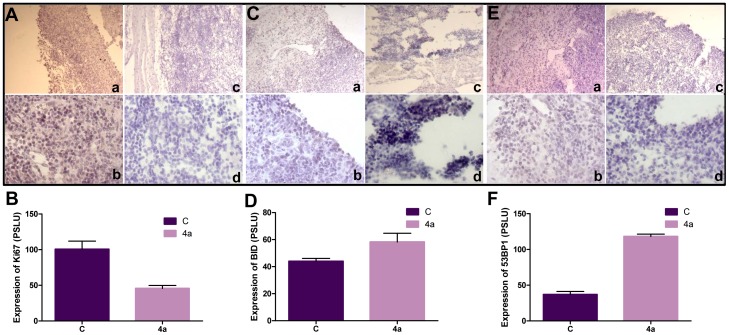
Immunostaining studies for apoptotic and DNA damage markers following treatment with 4a. **A–F.** Ki67, BID and 53BP1 immunostaining of tumor and treated tissues. The images were quantified using ImageJ software and standard error was plotted using independent images. **A, B.** Antibody staining for Ki67 on 25^th^ day tumor tissue (a, b) and tumor tissues treated with **4a** (c, d) and their quantification. **C, D**. Immunostaining for BID on 25^th^ day control tumor (a, b) and **4a** treated tumor (c,d) and their quantification. **E, F**. 53BP1 staining on 25^th^ day tumor tissue (a, b) and **4a** treated tumor tissue (c, d) and their quantification. Magnification of images shown in panels **a** and **c** are 10×, while **b** and **d** are 20×.

The Ki67 protein is expressed in all phases of the cell cycle except G0 and is considered as a marker for cellular proliferation [37], [38]. The tumor cell proliferation was investigated by immunohistochemical staining for Ki67 on tissue sections derived from untreated and **4a** treated tumors. Results showed efficient Ki67 and nuclear staining in tumor sections, while the number of Ki67 positive cells was substantially less in **4a** treated tumors (Fig. 7A, B). Further, we observed that the expression of p53 binding protein 1 (53BP1), and proapoptotic protein, BID was significantly high following treatment with **4a** in tumor tissues (25^th^ day of treatment) as compared to untreated tumor tissue (Fig. 7C–F), further suggesting the activation of apoptosis in tumor cells in mice. Therefore, our results show that **4a** treatment significantly inhibits tumor progression in mice.

**Figure 8:**
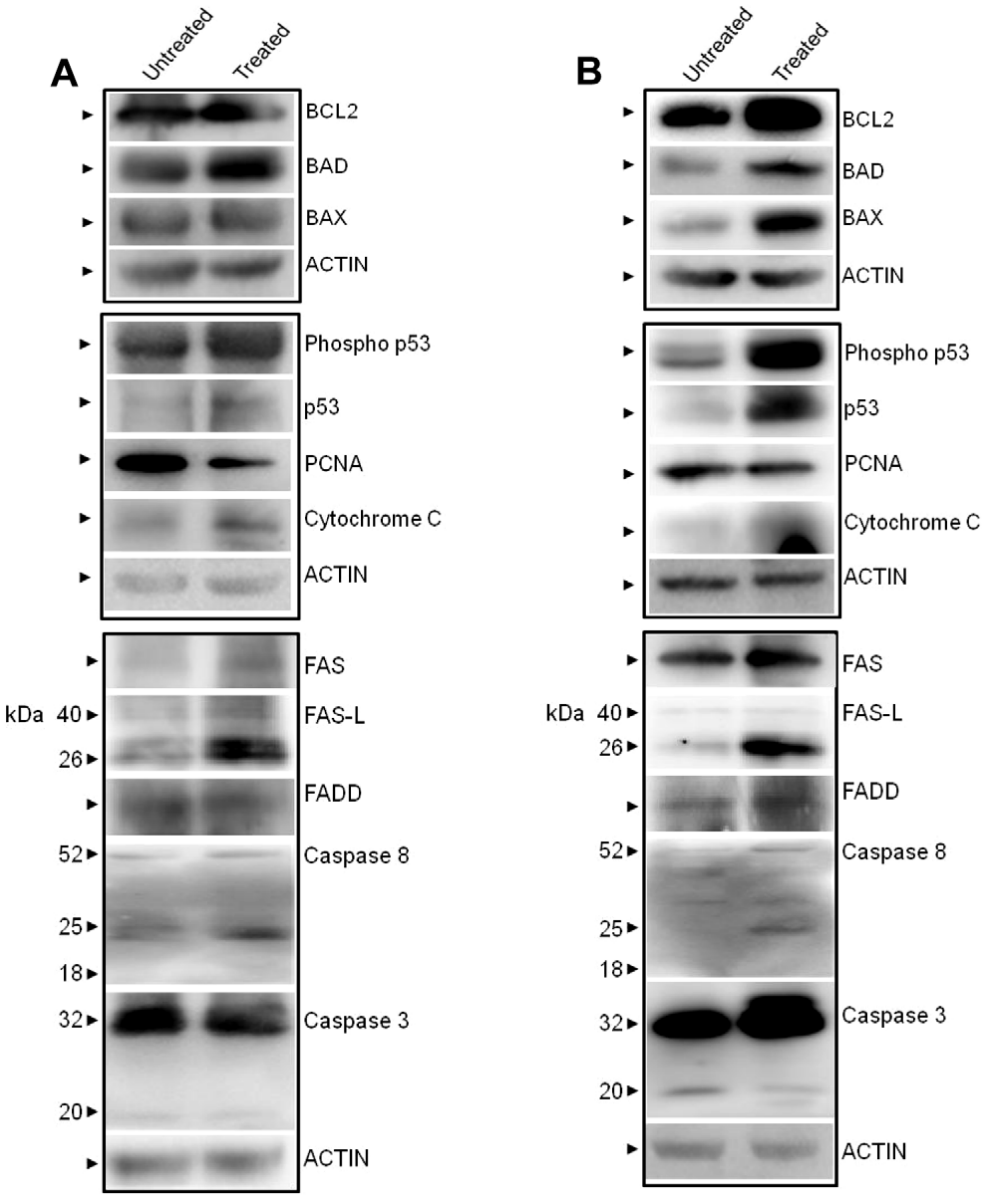
Comparison of expression of apoptotic proteins in 4a treated solid and liquid tumors in mice. **A. 4a** was orally administered to mice bearing solid tumor (6 doses, 20 mg/kg). Tumor tissues were collected after 25 days of **4a** treatment; lysate was prepared and used for western blotting. **B.** Expression of apoptotic proteins following **4a** treatment in liquid tumor. EAC cells were injected intraperitoneally in mice to generate liquid tumor. Following **4a** treatment (6 doses, 20 mg/kg) tumor cells were collected, lysate was prepared and used for western blotting. Antibodies used were BCL2, BAD, BAX, Phospho p53, p53, PCNA, CYTOCHROME C, FAS, FAS-L, FADD, CASPASE-8 and CASPASE-3. Actin was used as loading control (**A, B**).

Further, western blotting analysis was carried out on **4a** treated tumor cells from mice (both solid and liquid tumor) to evaluate the effect of **4a** on tumor progression (Fig. 8A, B). Results showed upregulation of proapoptotic proteins, BAD and BAX in both tumor models (Fig. 8A, B). We noted an upregulation of expression of BCL2, which needs to be studied further. A moderate downregulation of PCNA, a cell proliferation marker was also observed, which is consistent with immunohistochemistry results. Besides, we have also observed upregulation of both activated and normal p53, FAS, FAS-L, FADD and CYTOCHROME C (Fig. 8A, B) suggesting that the mechanism of cell death induced by **4a** in tumor tissues within the animals and cancer cell lines was comparable. We also observed cleavage of CASPASE-8 in both cases although CASPASE-3 cleavage was undetectable.

## Discussion

Synthesis and evaluation of promising novel anticancer compounds remains an important challenge for drug discovery [39]. Recently, we have synthesized and characterized a series of Levamisole derivatives and identified **4a** as the most potent molecule [21]. In the present study, we found that **4a** treatment resulted in efficient ROS production, which is an indicator of DNA damage. Further, we show that **4a** induces cytotoxicity by activating the extrinsic pathway of apoptosis.

EAC cells possessing malignant features of cancer are used commonly for inducing tumors in Swiss albino mice, and for evaluating anti-cancer activity of small molecules *in vivo* [26]–[28], [40], [41]. Our results show that **4a** treatment led to a significant reduction in tumor size. More than 4-fold increase in lifespan of treated mice was observed after **4a** treatment, when compared with untreated animals with tumor. Histological evaluation of tumor and normal tissues following compound treatment further indicates that its effect was mostly restricted to tumor cells. Thus, effectiveness of **4a** at low concentrations in mice makes it a potential cancer therapeutic agent.

Interestingly, Levamisole, the parental compound failed to show any cytotoxic or antitumor activity at concentrations equivalent to **4a**. There are contradicting reports on anticancer activity of Levamisole in the literature. In one of the studies, Levamisole failed to show any anticancer activity even at higher concentrations [42]. Howerever, other studies have reported that Levamisole can act as a potent anticancer drug in EAC as well as other cancer cell lines [18], [43], [44]. It has also been shown that Levamisole can act as immunomodulatory agent. Interestingly, it could enhance the effect of anticancer drugs such as chlorambucil, when used together, by acting as an immunostimulator [43]. Although combined therapy of Levamisole along with other anticancer agents increases sensitivity of Ehrlich ascites carcinoma, it has been demonstrated to have adverse effects on liver and kidney metabolism and pathology. In the present study also, we noticed hepatic abnormalities in case of Levamisole. On the other hand, **4a,** despite being a more potent anticancer compound had limited adverse effect on histopathology or metabolic functions of liver and kidney.

Immunohistochemical studies showed regression of tumor cell proliferation as evident by Ki67 stained cells following **4a** treatment, which was also consistent in case of western blot analysis, where we observed downregulation of PCNA after treatment with **4a** in tumor lysate. Elevated expression of proapoptotic protein BID and damage sensor 53BP1, were also observed in tumor treated tissues, suggesting the activation of apoptosis following **4a** treatment. These results suggest that **4a** treatment significantly inhibits tumor cell proliferation and increase the life span of **4a** treated mice.

p53 is one of the most well studied transcription factors that plays a critical role in cell cycle arrest, apoptosis and DNA repair in response to a variety of cellular stresses, including DNA damage [45], [46]. **4a** treatment resulted in a dose-dependent upregulation of p53, which could be a result of ROS-mediated disruption of mitochondrial membrane potential and DNA damage. p53 mediated transcriptional activation could regulate activation of pro-apoptotic protein BAX [47] which in turn changes the mitochondrial membrane potential resulting in the release of CYTOCHROME C [48], [49]. Based on our results, it is evident that overproduction of intracellular ROS, upregulation of p53 and release of CYTOCHROME C into cytosol, would result in the p53 mediated apoptosis. Further, p53 upregulation can modulate the expression of PUMA, a BCL2 family protein and an important mediator of p53-dependent apoptosis [50], [51]. Consistent with that we found an upregulation of PUMA, upon treatment with **4a** (Fig. 4A). Recently, a study showed necrotic mode of cell death by p53 under oxidative stress, independent of caspase cleavage. This study also showed release of CYTOCHROME C into the cytosol upon addition of p53 to purified mitochondria [52]. Although, **4a** could induce ROS production at early time points, its levels were not constant or maintained, and this transient ROS production did not result in necrosis. Instead, it led to phosphorylation of p53, cleavage of CASPASE-8 and CASPASE-3, further culminating in the activation of apoptosis.

Although, the level of cell survival protein, AKT and its phosphorylated form p-AKT, increased after treatment with **4a**, it failed to show any effect on survival of the cell. As described above, it is possible that upregulation of p53 and its phosphorylated form may be sufficient to overcome the effect due to AKT.

Consistent with the above conclusion, we observed that K562 cells were much less sensitive to **4a** with an IC_50_ value of 70, unlike the other three leukemic cell lines studied. Ours and other groups have shown that K562 does not express wild type p53 [53]–[55]. This suggests that in the absence of p53, **4a** is unable to induce a comparable level of apoptosis suggesting that it might act in a p53 dependent manner. However, this needs to be investigated further.

Generally during apoptosis, increase in proapoptotic proteins and decrease in the levels of antiapoptotic proteins are required for maintaining the ratio between them. However, upon addition of **4a,** we observed an interesting upregulation of antiapoptotic proteins leading to imbalance in the overall ratio and finally resulting into apoptosis. Previous studies have also reported an upregulation of BCL2 followed by activation of apoptosis [56], [57].

In the present study, we observed a dose dependent upregulation of FAS after **4a** treatment in both cell lines and mouse tumor models (Fig. 8). Induction of apoptosis through cell surface death receptors (FAS and FAS-L) results in the activation of an initiator, CASPASE-8. Activation of death receptors with their ligands provokes the recruitment of adaptor proteins, such as the FAS-associated death domain proteins (FADD), which in turn recruit and aggregate CASPASE-8, thereby promoting its auto processing and activation (Fig. 4D). Activated CASPASE-8 proteolytically processes and activates CASPASE-3 that culminates in substrate proteolysis leading to cell death. Consistent to this, we observed an upregulation of the death receptor proteins, FAS and FAS-L in CEM cells. Our results suggest that CASPASE-8 and CASPASE-3 cleavage in **4a** treated CEM cells could result in DNA fragmentation and apoptosis (Fig. 4D).Moreover, we observed cleavage of BID by CASPASE-8 into its truncated version t-BID which in turn facilitates the mitochondrial pathway of apoptosis (Fig. 4B). The mitochondrial protein, SMAC/DIABLO, plays an important role in apoptosis by eliminating the inhibitory effect of IAPs (inhibitor of apoptosis proteins) on caspases [58]. Our results show a dose dependent activation of SMAC/DIABLO upon treatment with **4a**.

**Figure 9:**
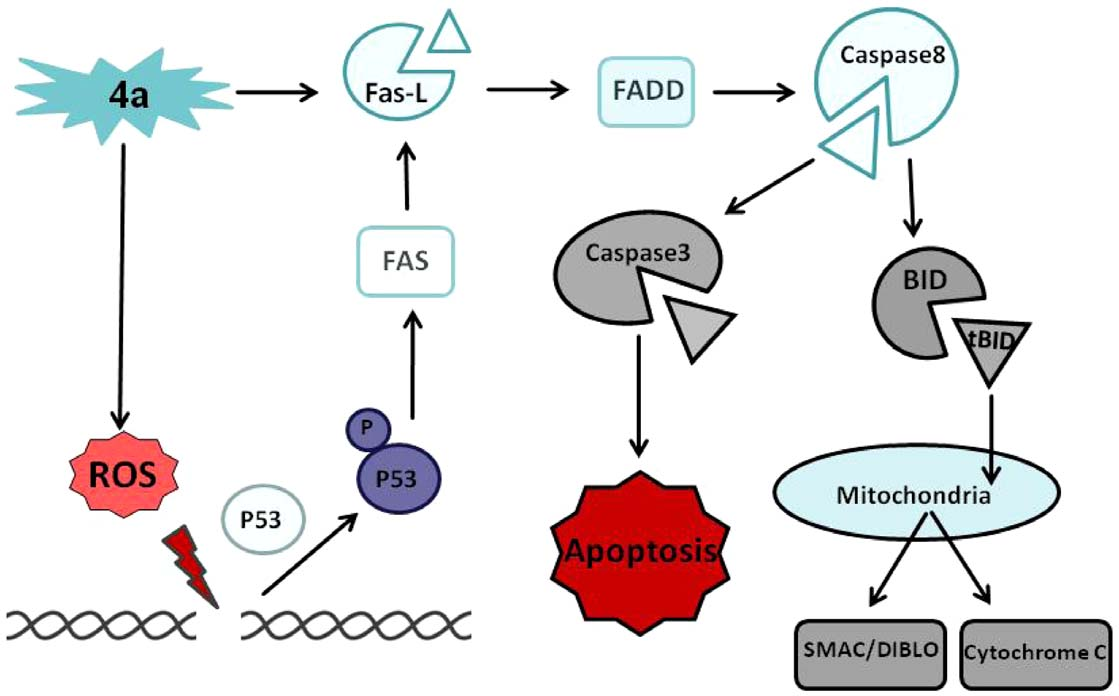
Proposed model for mechanism of 4a induced cytotoxicity by induction of apoptosis. **4a** treatment resulted in production of ROS, thereby damaging the DNA, which in turn helped in upregulation and phosphorylation of p53, where it activated extrinsic pathway of apoptosis by activating FAS, cleavage of FAS-L. These activated death receptors resulted in the recruitment of adaptor proteins, FAS-associated death domain proteins (FADD), which recruits and aggregates CASPASE-8, thereby promoting its auto processing and activation. Activated CASPASE-8 cleaves BID into t-BID, which further facilitates in the release of CYTOCHROME C from mitochondria, further cleaving PROCASPASE-3 into the effector CASPASE-3 which leads to cell death.

In summary, **4a** treatment resulted in an increase in DNA damage which led to the upregulation of p53. **4a** treatment activates FAS and FAS-L death receptor pathway, leading to cleavage of CASPASE-8 followed by activation of CASPASE-3 (Fig. 9). Thus, the extrinsic pathway of apoptosis is induced by **4a** leading to cell death both *in vivo* and *ex vivo* suggesting that **4a** could be used as a potential cancer therapeutic agent.

## Supporting Information

Figure S1 Lactate dehydrogenase release assay on **4a** treated CEM cells at different timepoints to evaluate the cell damage caused by **4a**.
(PPT)

Figure S2 Determination of ROS production following **4a** treatment on CEM cells at different timepoints.
(PPT)

Figure S3 Histological sections of thigh and liver tissues of **4a** treated and untreated mice.
(PPT)

Figure S4 Histological sections of liver and brain tissues of from **4a** or Levamisole treated normal mice.
(PPT)
