# Supplementary figures and images for: Novel Levamisole Derivative Induces Extrinsic Pathway of Apoptosis in Cancer Cells and Inhibits Tumor Progression in Mice
Source: PLoS One. 2012 Sep 10;7(9):e43632. doi: 10.1371/journal.pone.0043632 (PMC3438185; doi:10.1371/journal.pone.0043632)

## Slide 1
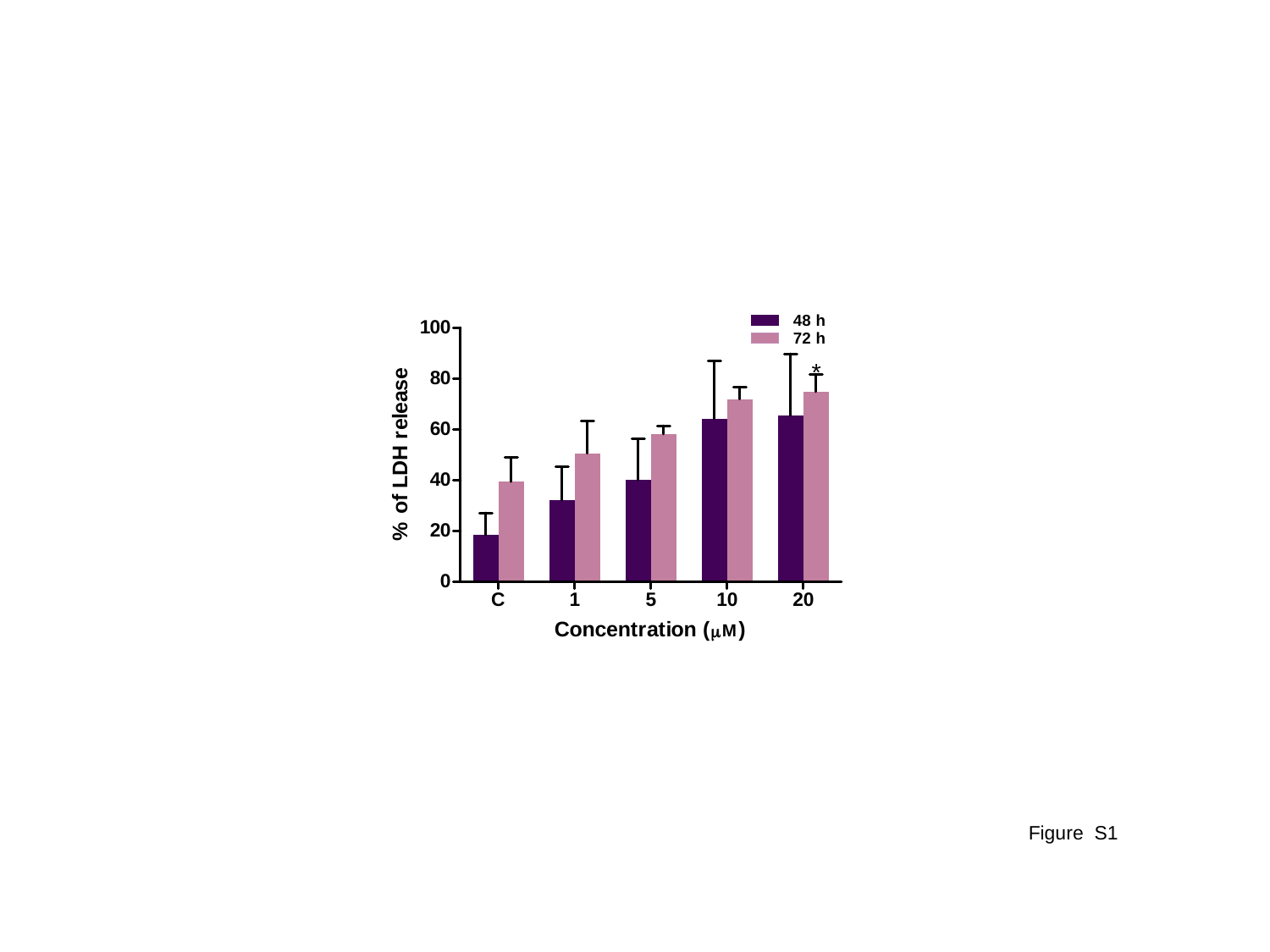

Figure S1

Supplement: Figure S1 — Lactate dehydrogenase release assay on 4a treated CEM cells at different timepoints to evaluate the cell damage caused by 4a. (PPT) [file pone.0043632.s001.ppt]

## Slide 1
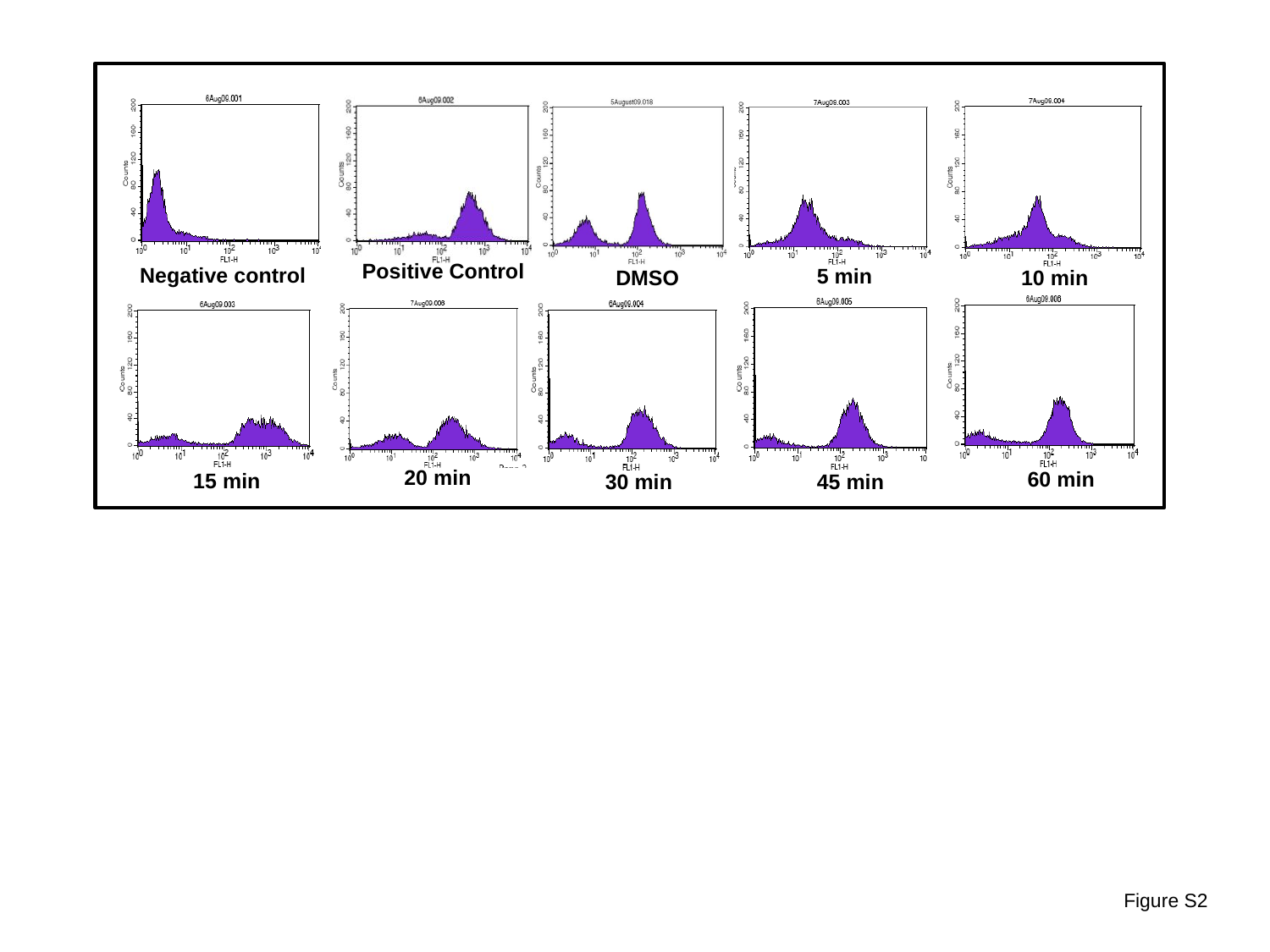

Positive Control
Negative control
5 min
DMSO
10 min
20 min
60 min
15 min
30 min
45 min
Figure S2

Supplement: Figure S2 — Determination of ROS production following 4a treatment on CEM cells at different timepoints. (PPT) [file pone.0043632.s002.ppt]

## Slide 1
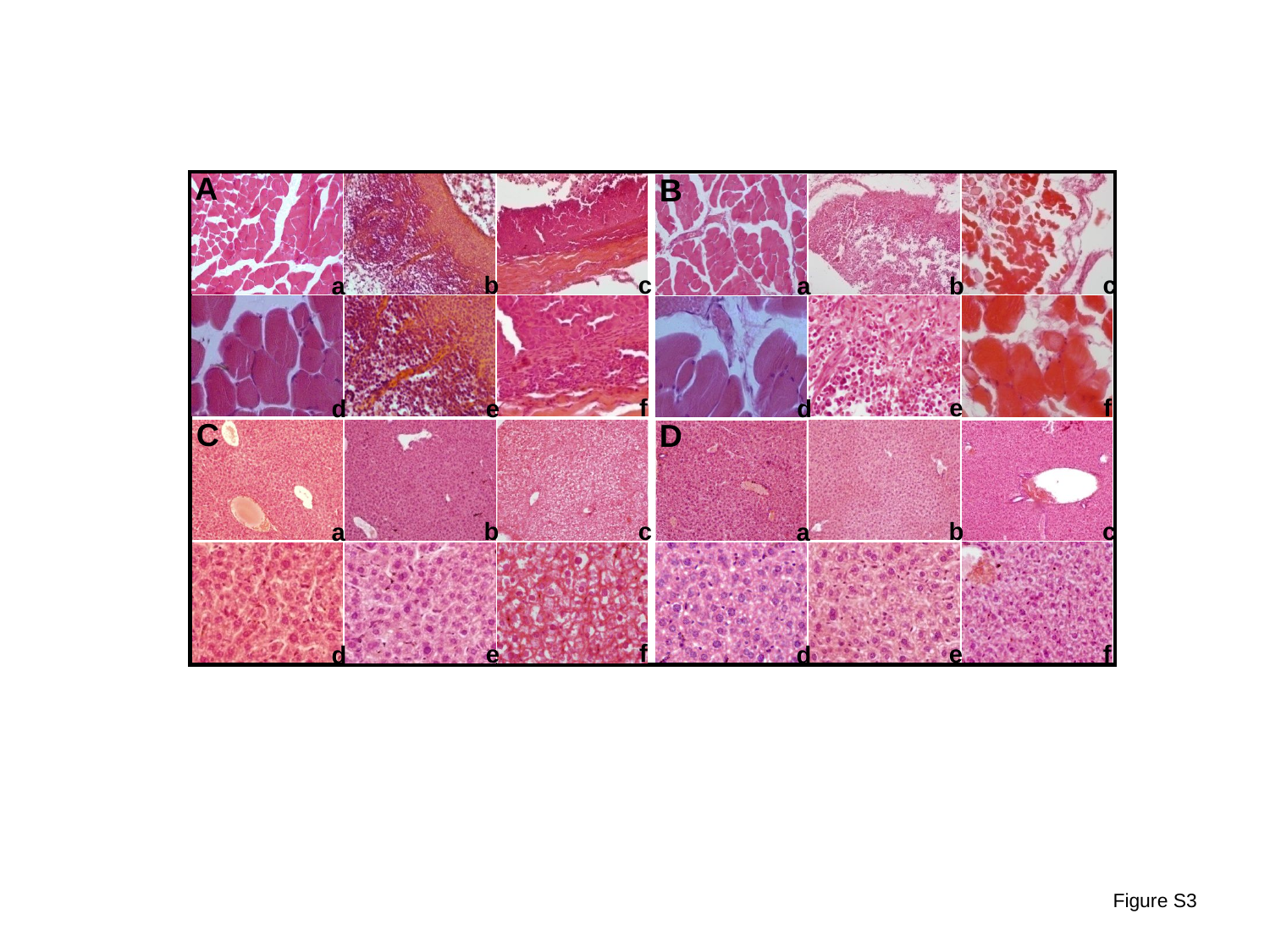

A
B
c
b
c
a
b
a
f
e
f
e
d
d
C
D
c
b
c
b
a
a
f
e
f
e
d
d
Figure S3

Supplement: Figure S3 — Histological sections of thigh and liver tissues of 4a treated and untreated mice. (PPT) [file pone.0043632.s003.ppt]

## Slide 1
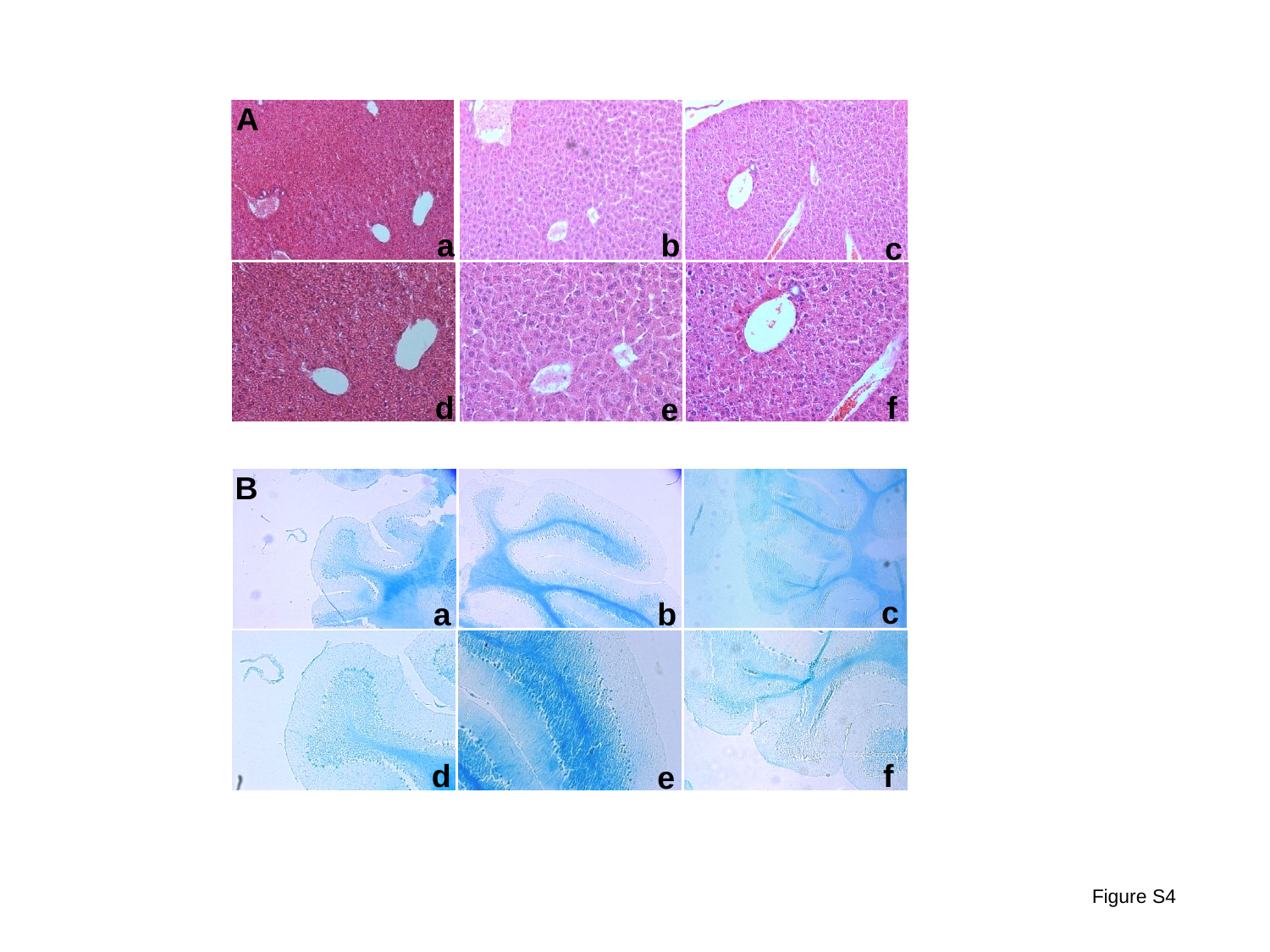

A
a
b
c
d
f
e
B
c
a
b
d
f
e
Figure S4

Supplement: Figure S4 — Histological sections of liver and brain tissues of from 4a or Levamisole treated normal mice. (PPT) [file pone.0043632.s004.ppt]
